# Labelling Selective Sweeps Used in Durum Wheat Breeding from a Diverse and Structured Panel of Landraces and Cultivars

**DOI:** 10.3390/biology10040258

**Published:** 2021-03-24

**Authors:** Jose Miguel Soriano, Carolina Sansaloni, Karim Ammar, Conxita Royo

**Affiliations:** 1 Sustainable Field Crops Programme, Institute for Food and Agricultural Research and Technology (IRTA), 25198 Lleida, Spain; conxita.royo@irta.cat; 2 Centro Internacional de Mejoramiento de Maíz y Trigo (CIMMYT), El Batán, Texcoco 56237, Mexico; c.sansaloni@cgiar.org (C.S.); k.ammar@cgiar.org (K.A.)

**Keywords:** Mediterranean basin, genetic diversity, marker trait association, gene flow, eigenGWAS

## Abstract

**Simple Summary:**

Evaluation of the genetic diversity of a crop species is a critical step for breeding. Landraces are essential to avoid genetic erosion, and Mediterranean landraces are an important group of genetic resources due to their high genetic variability, adaptation to local conditions in rainfed environments, and their resilience to pests and pathogens. This study uses a genome-wide association approach employing eigenvectors to identify selective sweeps among Mediterranean durum wheat landraces and a world panel of modern cultivars.

**Abstract:**

A panel of 387 durum wheat genotypes including Mediterranean landraces and modern cultivars was characterized with 46,161 diversity arrays technology (DArTseq) markers. Analysis of population structure uncovered the existence of five subpopulations (SP) related to the pattern of migration of durum wheat from the domestication area to the west of the Mediterranean basin (SPs 1, 2, and 3) and further improved germplasm (SPs 4 and 5). The total genetic diversity (*H_T_*) was 0.40 with a genetic differentiation (*G_ST_*) of 0.08 and a mean gene flow among SPs of 6.02. The lowest gene flow was detected between SP 1 (presumably the ancient genetic pool of the panel) and SPs 4 and 5. However, gene flow from SP 2 to modern cultivars was much higher. The highest gene flow was detected between SP 3 (western Mediterranean germplasm) and SP 5 (North American and European cultivars). A genome wide association study (GWAS) approach using the top ten eigenvectors as phenotypic data revealed the presence of 89 selective sweeps, represented as quantitative trait loci (QTL) hotspots, widely distributed across the durum wheat genome. A principal component analysis (PCoA) using 147 markers with −log_10_ *p* > 5 identified three regions located on chromosomes 2A, 2B and 3A as the main drivers for differentiation of Mediterranean landraces. Gene flow between SPs offers clues regarding the putative use of Mediterranean old durum germplasm by the breeding programs represented in the structure analysis. EigenGWAS identified selective sweeps among landraces and modern cultivars. The analysis of the corresponding genomic regions in the ‘Zavitan’, ‘Svevo’ and ‘Chinese Spring’ genomes discovered the presence of important functional genes including *Ppd*, *Vrn*, *Rht*, and gene models involved in important biological processes including LRR-RLK, MADS-box, NAC, and F-box.

## 1. Introduction

Durum wheat (*Triticum turgidum* L. var. *durum*) originated in the Fertile Crescent 10,000 years ago and propagated across the Mediterranean basin, arriving in the Iberian Peninsula from two routes: southern Europe and northern Africa [1,2]. During this migration, both natural and human selection occurred and new traits allowing adaptation to the new environments were selected, resulting in the expansion of local landraces [3]. Landraces were broadly cultivated until the 1960s, when they were replaced by new and improved semi-dwarf cultivars arising from the Green Revolution. However, due to their wide genetic diversity, landraces are key for avoiding genetic erosion [4] and are valuable for crop breeding, providing new alleles for the improvement of important agronomic traits. Mediterranean landraces are a valuable group of genetic resources due to their adaptation to their regions of origin, their huge genetic diversity [5,6], their resilience to abiotic stresses [7], and their resistance to pests and diseases [8,9,10]. Natural and artificial selection result in adaptive changes to the populations that can be followed at the allele level by the identification of loci under selection [11]. Identification of loci under selection has been performed classically by different methods, including the genetic differentiation (F_ST_) scan that has been widely applied in crops [12]. Recently, Chen et al. [13] developed a single-marker regression approach based on principal component analysis (PCA), eigenGWAS. In this approach, similar to classical GWAS, the phenotype is substituted with the eigenvectors to use the genetic variation in the population to identify selection signals. EigenGWAS has been successfully applied in crop species such as maize [14], wheat [15], and barley [16].

In the last few years, high-throughput genotyping technologies such as single nucleotide polymorphism (SNP) arrays and genotyping by sequencing (GBS) platforms, including diversity arrays technology (DArTseq), have been widely used in wheat to identify marker–trait associations (MTAs) in highly saturated maps [17,18,19,20]. Additionally, the progress in whole genome sequencing of emmer wheat [21], wheat [22], and durum wheat [23] allows for the understanding of the genetic diversity and adaptation patterns in wheat, as well as the discovery of genes of interest for breeding.

The main objectives of the current study were: (a) to analyze the genetic diversity and population structure in a GWAS panel, including Mediterranean durum wheat landraces and modern cultivars from the main durum wheat-growing regions in the world; and (b) to identify the selection patterns in the durum wheat genome driving the differentiation among Mediterranean landraces and modern cultivars.

## 2. Materials and Methods

### 2.1. Plant Material

The diversity panel was comprised of a panel of 387 durum wheat genotypes, including 183 landraces from 24 Mediterranean and eastern European countries and a set of commercial varieties from 24 countries, representing the main durum wheat growing areas in the world (204 genotypes) (Supplementary Table S1). The landrace populations were supplied by public gene banks (the Centro de Recursos Fitogenéticos CRF-INIA, Spain, the ICARDA Germplasm Bank, and the USDA Germplasm Bank) and were increased in bulk and purified to select the dominant type (frequency higher than 80%). Modern cultivars were provided by the IRTA durum wheat collection, international centres (CIMMYT and ICARDA), and breeding companies.

### 2.2. Genotyping

DNA was isolated from fresh leaf samples according to Doyle and Doyle [24]. High-throughput genotyping was performed at Diversity Arrays Technology Pty Ltd. (Canberra, Australia) (http://www.diversityarrays.com, accessed on 1 February 2020) with the DArTseq GBS platform [25]. A total of 46,161 markers were used to genotype the association mapping panel, including 35,837 presence-absence variants (PAV) and 10,324 SNPs. The consensus map of wheat v4, available at https://www.diversityarrays.com/technology-and-resources/genetic-maps/ (accessed on 1 February 2020) (Diversity Arrays Technology Pty Ltd., Canberra, Australia), was used for mapping purposes.

### 2.3. Data Analysis

Polymorphic information content (PIC) values were calculated using Cervus software v3.0.7 [26]. Genetic diversity was estimated as total diversity (*H_T_*) [27] using Arlequin 3.5.2.2 [28]. The coefficient of genetic differentiation (*G_ST_*) was calculated as *G_ST_ = D_ST_/H_T_*, where *D_ST_* is the genetic diversity between populations, calculated as *D_ST_* = *HT − HS*, with H_S_ as the mean genetic diversity within populations. Gene flow was estimated as Nm = 0.5 (1 − *G_ST_*)/*G_ST_* according to McDonald and McDermott [29].

Linkage disequilibrium (LD) was estimated using TASSEL 5.0 [30] as the square of marker correlations (*r*^2^) for mapped markers at a significance level of *p* < 0.001 with a sliding window of 50 cM. The *r*^2^ values were plotted against the genetic distance and a locally estimated scatterplot smoothing (LOESS) curve was fitted to determine the distance at which the curve intercepts the line of a critical value of *r*^2^ to estimate the LD decay. The critical value of *r*^2^ was determined as the mean *r*^2^ for each genome.

The genetic structure of the association mapping panel was estimated using the Bayesian clustering algorithm implemented in the software STRUCTURE v2.3.4 [31], which uses an admixture model with burn-in and Monte Carlo Markov chain for 10,000 and 100,000 cycles, respectively. The Evanno method [32] was used to calculate the most likely number of subpopulations using the online software STRUCTURE HARVESTER [33]. Principal coordinates analysis (PCoA) based on genetic distance was calculated using GenAlEx 6.5 [34]. Diversity analysis between genotypes was defined by the simple matching coefficient [35] using DARwin software v.6 [36]. The un-rooted tree was calculated using the neighbor-joining method [37].

### 2.4. Identification of Selective Sweeps

Identification of loci under selection among landraces and modern cultivars was performed by GWAS utilizing the eigenvectors corresponding to the top ten eigenvalues as the phenotype data, similar to the eigenGWAS [13], but using a mixed linear model (MLM) with TASSEL software version 5.0 [29]. The MLM accounted for population structure using a principal component analysis (PCA) matrix with 6 principal components as the fixed effect and a kinship (K) matrix as the random effect (PCA + K) at the optimum compression level. MLM followed the equation:

y = Xβ + Zu + e

where y is the trait value (the eigenvector in this case), β is the fixed effect for the marker, and u is a vector of random effects not associated with the markers; X and Z are incidence matrices linking y to β and u. Finally, e is the undetected vector of the random residual. In addition, the heading date was incorporated as a cofactor in the analysis. Two thresholds were established for considering marker–trait association (MTA) significance. A highly significant threshold was established using a false discovery rate (FDR) threshold [38] at *p* < 0.05, and a moderate threshold at −log_10_ *p* = 3. In order to simplify the GWAS results, QTL hotspots grouping closely located MTAs were determined based on LD decay. Graphical representations of Manhattan plots were carried out using the R package “CMplot” (http://www.r-project.org (accessed on 15 April 2020)).

### 2.5. Gene Annotation

Gene models for QTL hotspots were identified using the high-confidence gene annotation for the bread wheat genome reference sequence at https://wheat-urgi.versailles.inra.fr/Seq-Repository/Assemblies (accessed on 27 August 2020), the durum wheat reference sequence at https://wheat.pw.usda.gov/GG3/jbrowse_Durum_Svevo (accessed on 27 August 2020), and the wild emmer reference sequence of ‘Zavitan’ at https://wheat.pw.usda.gov/GG3/jbrowse_Zavitan (accessed on 27 August 2020).

## 3. Results

### 3.1. Genetic Diversity and Population Structure

Overall, 46,161 DArTseq markers were used to genotype the set of 387 durum wheat genotypes, of which 183 corresponded to Mediterranean and eastern Europe landraces and 204 to modern cultivars. To diminish the risk of false positives, markers and accessions were analyzed for the presence of duplicated patterns and missing values.

Of the 35,837 presence/absence variants (PAV), 24,188 had a known map position in the wheat v4 consensus map (Diversity Arrays Pty Ltd., Canberra, Australia). Of these, 4745 markers with a minor allele frequency (MAF) lower than 5% were excluded from the analysis, resulting in 19,443 PAVs remaining. Of 10,324 SNPs, 6957 were located on the wheat v4 consensus map. Of these, 1260 markers with missing data higher than 30% and 1011 markers with MAF < 5% were excluded from the analysis, resulting in a total of 4686 SNPs. Moreover, 413 markers were found to be duplicated among SNPs and PAVs, so the corresponding PAVs were discarded, leaving a total of 23,716 markers for the analyses. Forty-one percent of the markers corresponded to genome A and 59% to genome B. The total length of the map was 2129.2 cM, with a mean coverage of 11 markers/cM. Polymorphic information content (PIC) values were estimated for each chromosome, ranging from 0.26 in chromosome 7A to 0.29 in 7B, with an average of 0.28. PIC values showed a skewed distribution, with 48% of the markers having a PIC of <0.3 (Supplementary Figure S1).

**Figure 1:**
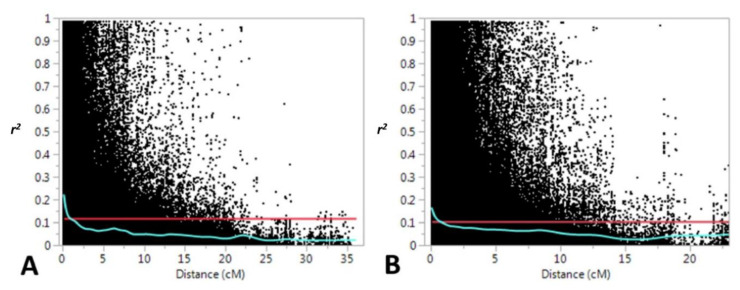
Linkage disequilibrium plots for (**A**) genome A; (**B)** genome B. The locally estimated scatterplot smoothing (LOESS) curve is represented in blue. The red line represents the mean value for the square of marker correlations (*r*^2^).

Linkage disequilibrium (*r*^2^) was estimated for locus pairs in genomes A and B. A total of 471,319 and 681,389 possible pair-wise loci were found for genomes A and B, respectively. The percentage of locus pairs showing LD at *p* < 0.001 was 43% for both genomes. Mean values for *r*^2^ were 0.12 and 0.11 for genomes A and B, respectively. These means were used as a threshold for estimating the intercept of the LOESS curve to determine the distance at which LD decays in each genome. LD decays were established at 1 cM for both genomes (Figure 1).

**Figure 2:**
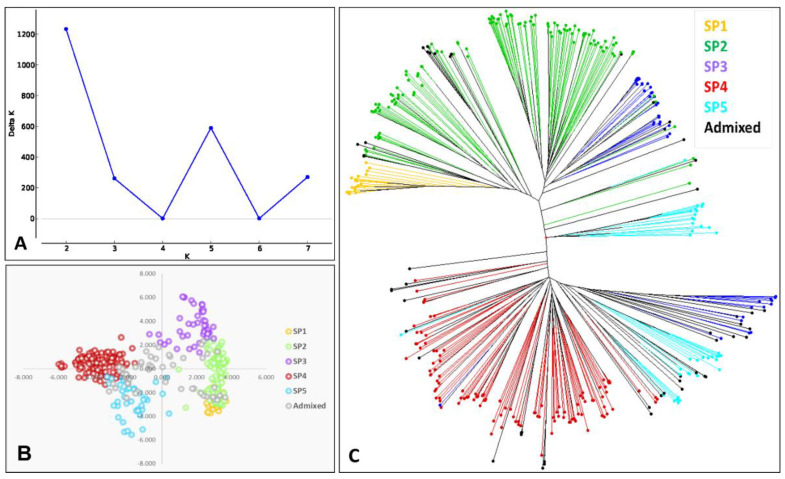
Genetic structure of the durum wheat collection. (**A**) Estimation of the number of subpopulations (SPs) according to the Evanno test. (**B**) Principal coordinates analysis (PCoA) based on genetic distance. (**C**) Unrooted neighbor-joining dendrogram.

Analysis of population structure was performed according to the distance of LD decay using only SNP markers showing less than 25% of missing data, minor allele frequencies higher than 10%, and PIC values higher than 0.3. A total of 1695 markers were used. The highest value for ΔK was observed for K = 2, followed by K = 5 (Figure 2A). In the first case, the Bayesian clustering method used the Evanno test [32] to separate the genotypes by their origin (landraces vs. modern cultivars). Considering a membership coefficient of *q* > 0.6, the first group comprised 201 genotypes, 19 of them modern cultivars (9%) and 182 (91%) landraces. The second group included 160 modern cultivars. Finally, 26 genotypes remained as admixed (one landrace and 25 modern cultivars). When K = 5, the genotypes were structured according to their origin, showing a geographical pattern. In this case, *q* > 0.5 was established as a threshold for considering a genotype within a subpopulation (SP).

The first group (SP 1) included 19 landraces, from which 89% corresponded to eastern Mediterranean countries and 11% to northern Mediterranean countries. The second group (SP 2) grouped 116 landraces and three modern cultivars. Landraces were mainly from northern Mediterranean countries (66%), and in lower percentages from eastern Mediterranean (21%) and southern Mediterranean (North of Africa) (13%) countries. The modern cultivars came from Italy (‘Creso’) and Spain (‘Anibal’ and ‘Paramo’). The third group (SP 3) showed both landraces (31) and modern cultivars (12), mainly from western Mediterranean countries (including the south of Europe and north of Africa) (84% and 83% of the landraces and modern cultivars, respectively). The fourth (SP 4) and fifth (SP 5) groups included only modern cultivars. SP 4 (116 genotypes) was represented by modern cultivars mainly developed from CIMMYT and ICARDA germplasm, whereas SP 5 (39 genotypes) represented modern cultivars mainly from northern America (56%) and Europe (France, Italy and Spain) (41%). The remaining 51 genotypes (17 landraces and 34 modern cultivars) remained as admixed.

A principal coordinate analysis (PCoA) was carried out to graphically represent the results of the structure analysis in a bi-dimensional plot (Figure 2B). In agreement with the structure analysis, the first two coordinates of the PCoA separated landraces, located on the positive side of the first coordinate, from the modern cultivars, located on the negative side of the first coordinate. Admixed genotypes were in the center of the plot. Within these clusters, the different subpopulations were clearly defined, as shown in Figure 2B.

As a complementary approach, a neighbor-joining tree based on a distance matrix was constructed to support the previous results (Figure 2C). The tree presented a main division in two clusters, grouping landraces and modern cultivars separately. Within the cluster of landraces, there is a clear separation among SP 1 with landraces from eastern Mediterranean countries, SP 2 with landraces from northern Mediterranean countries, and the western Mediterranean landraces from SP 3. This cluster, grouping western Mediterranean landraces and modern cultivars by structure analysis, separated both types of genotypes in the main clusters. The modern cluster separately grouped the genotypes from the western Mediterranean (SP 3), north America (SP 5) and cultivars developed by CIMMYT and ICARDA breeding programs (SP 4). In addition to these main clusters, a small one representing modern cultivars from north America and southern Europe remained separate.

Results of the analysis of molecular variance (AMOVA) indicated that variation within SPs accounted for 92% of the total variance, whereas the remaining 8% corresponded to variation between SPs. Total genetic diversity (*H_T_*) among SPs ranged from 0.40 in SP 4 to 0.35 for SP 3 and the admixed genotypes (Table 1). The genetic diversity among SPs (*D_ST_*) was low (0.03), causing a genetic differentiation (*G_ST_*) among SPs of 0.08. This means that only about 8% of the variability observed was due to differences between SPs, as previously reported by AMOVA. The estimation of the gene flow (*Nm*) among SPs was 6.02, indicating a high level of gene exchange according to the low genetic differentiation among the SPs. Comparisons among SPs revealed that gene flow ranged from 2.54 between SP 4 (modern cultivars mainly developed by CIMMYT and ICARDA) and SP 5 (modern cultivars from north America and Europe) to 69.81 between SP 2 (landraces mainly from northern Mediterranean countries) and SP 3 (western Mediterranean landraces and cultivars) (Table 1).

### 3.2. Identification of Loci under Selection by EigenGWAS

**Figure 3:**
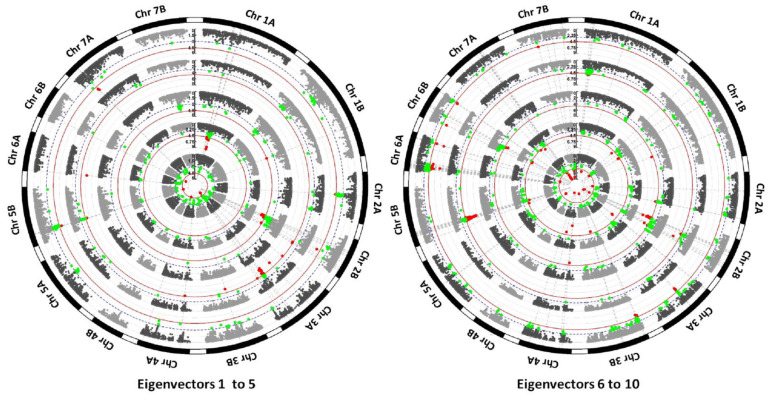
EigenGWAS for the top ten eigenvectors. Left circle: from the inside out, eigenvectors 1 to 5. Right circle: from the inside out, eigenvectors 6 to 10. Green dots correspond to significant marker–trait associations (MTAs) at a moderate threshold (−log_10_ *p* = 3.0, blue dotted line) and red dots correspond to significant MTAs above the false discovery rate (FDR) threshold (−log_10_ *p* = 4.6, red line).

EigenGWAS was conducted using the top ten eigenvectors resulting from the PCoA obtained for the whole collection of genotypes, including landraces and modern cultivars. The largest eigenvalue was 3600.4, explaining 11.3% of the genetic variation, whereas the 10th eigenvalue was 408.0, explaining 1.3% of the genetic variation. The top ten eigenvalues accounted for 32.3% of the genetic variation, which indicates the complexity of the population structure of this durum wheat collection. A total of 1575 marker–trait associations (MTAs) were reported for the top ten eigenvectors using a moderate threshold of −log_10_ *p* = 3.0. Based on the LD decay for a maximum distance of 1 cM, a highly significant FDR threshold at *p* < 0.05 was established for −log_10_ *p* = 4.6. Following this approach, 250 MTAs were significant (Figure 3, Supplementary Table S2, Supplementary Figure S2). The number of MTAs per eigenvector ranged from 57 for eigenvector 2 to 304 for eigenvector 10. Chromosome 2B showed the maximum number of associations (279), whereas chromosome 4B showed the lowest (10). The mean percentage of variance explained (*r*^2^) per MTA ranged from 0.003 to 0.108, with an average of 0.034.

To simplify this information and to identify consensus genomic regions controlling loci under selection, QTL hotspots were identified by grouping closely located MTAs. Confidence intervals were defined based on the distance of 1 cM of the LD decay. A total of 89 QTL hotspots were identified, including 1491 MTAs, 248 of them (17%) above the FDR threshold (Table 2). The remaining 84 single MTAs were not considered further in the analysis. The number of MTAs per QTL hotspot ranged from 2 to 158, with a mean of 17 MTAs/QTL hotspot. The number of QTL hotspots per chromosome ranged from two in chromosome 4B to nine in chromosome 3A. The number of MTAs per chromosome ranged from 7 in chromosome 4B to 277 in chromosome 2B. Chromosome 4B did not carry any MTA above the FDR threshold, whereas chromosome 5B reported 51 MTAs out of 184 above the FDR threshold.

### 3.3. Identification of Selection Regions among SPs

**Figure 4:**
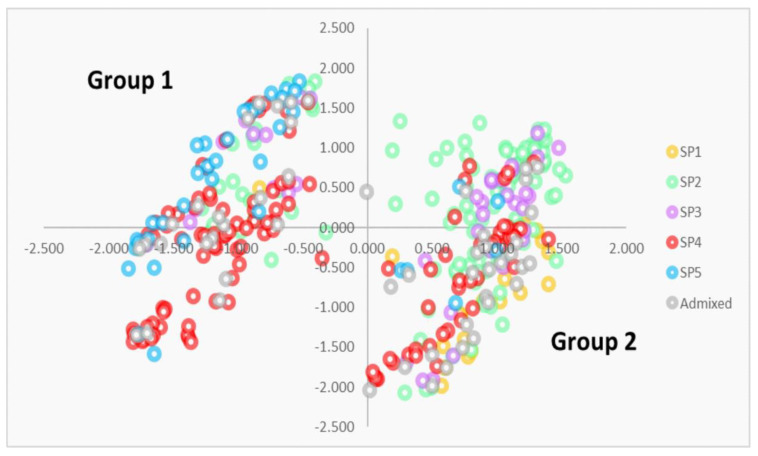
PCoA derived from the markers with −log_10_ *p* > 5 in the eigenGWAS.

To identify the genome regions most involved in the selection among the different SPs, markers with −log_10_ *p* >5 (147) from the eigenGWAS were selected and a PCoA was carried out (Figure 4). Markers were widely distributed along the genomes in all chromosomes, except chromosome 4B which harbored at least two MTAs or one QTL hotspot.

The PCoA separated two clear groups: group 1, on the left of the *Y*-axis, included 173 genotypes (mainly modern cultivars (79%)), whereas group 2, on the right of the *Y*-axis, included 214 genotypes, of which 69% corresponded to landraces. By SPs, those represented mainly by landraces (SP 1, SP 2 and SP 3) were mostly included in group 2 (95%, 77%, and 63%, respectively), whereas SPs represented mainly by modern cultivars (SP 4 and SP 5), were mostly included in group 1 (63% and 85%, respectively). All north American cultivars from SP 5 were located within group 1. Most of the landraces from the northern Mediterranean included in SP 2 were also represented in group 1.

The selected 147 markers, corresponding to 35 QTL hotspots, were analyzed to identify differences in the marker allele between both groups, as well as the different SPs (Supplementary Table S3). To identify robust differences among groups, a threshold of allele frequency within a group was established at 80%. When both alleles of the marker comply with this condition, the marker was considered significant for locus selection. Following this approach, 35 markers from five QTL hotspots were identified: eigenQTL2A.7, eigenQTL2B.3, eigenQTL3A.5, eigenQTL3A.6 and eigenQTL3A.7 (Table 3). However, when the markers were blasted against the reference genomes of bread wheat [22], durum wheat [23], and wild emmer [21], it was observed that markers corresponding to eigenQTL3A.5, eigenQTL3A.6, and eigenQTL3A.7 shared the same physical positions.

### 3.4. Gene Annotation

**Figure 5:**
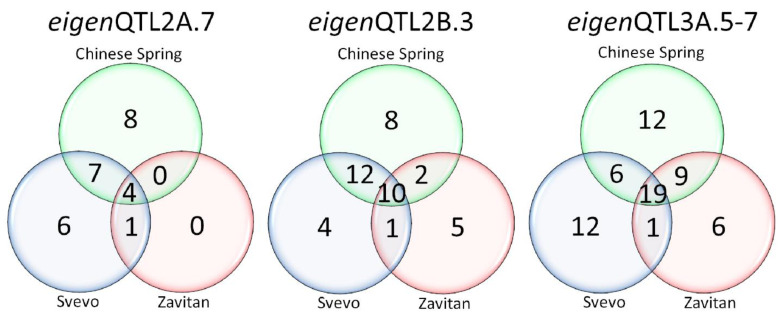
Comparison of unique gene models among the different genomes within the selected QTL hotspots.

Gene models were successfully identified using the different Gbrowse tools for the bread wheat cultivar ‘Chinese Spring’ [22], the durum wheat cultivar ‘Svevo’ [23], and the wild emmer cultivar ‘Zavitan’ [21] (Supplementary Table S4). The genome interval to identify gene models was defined based on the position of the flanking markers of the corresponding QTL hotspot. Thus, for eigenQTLT2A.7, 27, 29, and 6 gene models were identified in 1.40 Mb, 1.67 Mb and 270 Kb for ‘Chinese Spring’, ‘Svevo’ and ‘Zavitan’, respectively. For eigenQTLT2B.3, 47, 36, and 23 gene models, with 3.92 Mb 3.79 Mb and 4.11 Mb in ‘Chinese Spring’, ‘Svevo’ and ‘Zavitan’, respectively. Finally, eigenQTLT3A.5–7 were those with a higher number of gene models for the three genomes, with 77, 62, and 42 covering 6.12 Mb, 6.73 Mb and 6.57 Mb, respectively. Some of the gene models were represented in clusters, as was the case for F-box proteins, kinase proteins and resistance genes (Supplementary Table S4). Figure 5 summarizes the identification of common gene models between the three genomes for each of the three selected QTL hotspots. To reduce complexity, when a gene model was represented by more than one copy, it was reduced to a unique gene.

From 133 gene models within the three QTL hotspots, 33 (25%) were common for the three genomes, 25 (19%) were common between ‘Chinese Spring’ and ‘Svevo’, 11 (8%) were common between ‘Chinese Spring’ and ‘Zavitan’, and 3 (2%) were in common between ‘Svevo’ and ‘Zavitan’. Finally, 46% of the gene models were unique for the different genomes.

## 4. Discussion

### 4.1. Genetic Diversity and Population Structure

Genetic diversity is essential in plant breeding because it represents a source of new alleles for genes of interest. A useful approach for recovering and broadening allelic variation in traits of interest is the use of landraces in breeding programs [40], which may be of particular interest for suboptimal environments such as those prevailing in the Mediterranean basin [41].

The average chromosomal PIC value was 0.28. This value is similar to that reported previously in studies using bi-allelic markers such as SNP or DArT in durum wheat. Baloch et al. [42] reported PIC values of 0.26 and 0.30 depending on the marker type, (DArTseq or SNP, respectively). Kabbaj et al. [43] found a PIC value of 0.32 with 8173 SNPs from the Axiom 35K array. Pascual et al. [39] using a collection of Spanish landraces of bread and durum wheat genotyped with the DArTseq technology and obtained an average PIC value for both species between 0.30 and 0.35. According to the classification proposed by Botstein et al. [44] which separates PIC values into three categories of highly informative (PIC > 0.5), moderately informative (0.25 < PIC < 0.5) and slightly informative (PIC < 0.25), the markers in our panel are considered moderately informative. In previous studies from our group in durum and bread wheat, Soriano et al. [45] genotyped a panel of 192 durum wheat genotypes (mainly Mediterranean landraces) with 44 microsatellite markers and found an expected heterozygosity of 0.71. Rufo et al. [46] genotyped bread wheat collections of landraces and modern cultivars with a 15K SNP array and obtained a mean PIC value of 0.30, in accordance with the results obtained in the present study. These lower PIC values using DArTseq are explained by their bi-allelic nature, as the maximum PIC corresponds to 0.5 when both alleles have the same frequency [47].

Population structure analysis clearly divided the collection into two main subpopulations based on historical breeding periods, separating the genotypes in landraces and modern cultivars. To conduct a deeper analysis, the second highest value for K in the Evanno test was used. The genetic distribution of the landraces in the three SPs and the huge gene flow between them may be associated with the pattern of migration of durum wheat from the Fertile Crescent to the west of the Mediterranean basin described by Moragues et al. [48]. SP 1 contains the largest proportion of landraces from countries close to the zone of wheat domestication (89.4%), and only two Italian landraces (10.5%). Therefore, it is conceivable that SP 1 may putatively incorporate the oldest genetic background within the germplasm panel used in this study. The lowest level of admixture in SP 1 (89% of the genotypes with *q* > 0.7) agrees with this hypothesis. SP 2 could represent a further step in the history of wheat dispersal within the Mediterranean basin, as it gathers 21% of landraces from eastern Mediterranean countries, but 76% from western areas where it is supposed that wheat arrived between 2 and 3 millennia after its domestication [1]. Finally, SP 3 includes 72% of landraces and 28% of modern cultivars from western Mediterranean countries, the most distant from the area of wheat domestication, and so the most evolved from an evolutionary point of view. The highest gene flow between SP 2 and SP 3 and the lower, but still very high gene flow between SP 1 and SP 2, agree with this interpretation.

Gene flow between SPs offers clues regarding the putative use of Mediterranean old durum germplasm by the breeding programs represented here. The lowest gene flow was detected between SP 1 (assumed to gather the ancient genetic pool of the panel) and SPs involving only modern germplasms (SP 4 and SP 5). However, gene flow from SP 2 to modern cultivars was much higher, in agreement with the fact that this SP includes landraces adapted to a wide range of environmental conditions. The highest gene flow between SP 3 and SP 5 suggests that modern north American and European cultivars incorporate a significant portion of the genetic background of germplasms adapted to western Mediterranean environments. The relatively low gene flow observed between Mediterranean germplasms and the CIMMYT–ICARDA genetic pool may be a consequence of these international centers acting globally, thus incorporating germplasms in their breeding programs from around the world. SP 4 and SP 5 included only modern cultivars and had a low genetic flow between them, in agreement with the CIMMYT and north American durum wheats belonging to different germplasm pools [49,50].

Modern SPs presented a higher genetic diversity than SPs that included landraces in the following direction: SP 4 > SP 5 > SP 1 = SP 2 > SP 3. In agreement with the international nature of CIMMYT and ICARDA and their role as germplasm providers worldwide, SP 4 incorporates a wide range of cultivars with a worldwide distribution, thus showing a heterogeneous genetic background and the largest genetic diversity. SP 2 and SP 3 have mainly a western Mediterranean background (including the south of Europe and the north of Africa) and thus, with higher germplasm exchange, they could produce uniformity in the cultivars. The slightly higher values of *H_T_* observed in modern SPs may be due to the type of markers used in the study, as DArTseq and SNP are biallelic markers. For example, Soriano et al. [45] used SSR markers in a similar collection of 172 Mediterranean landraces and 20 modern cultivars and found higher values for *H_T_* in landrace SP and lower numbers of alleles in modern cultivars.

Genetic differentiation indicated that only 8% of the variability observed corresponded to differences between SPs, according to the high estimation of gene flow among SPs, thus indicating a high level of genetic exchange. Comparison of the genetic exchange between SPs revealed that the highest gene flow was observed between the western Mediterranean landraces and modern cultivars from western Mediterranean countries, as well as those between landraces from east to west in the Mediterranean basin. However, the lowest gene flow was found between eastern Mediterranean landraces and the germplasms from CIMMYT and ICARDA and between these germplasms and the modern cultivars from north America. This agrees with the results reported by Parzies et al. [51] and Ben-Romdhane et al. [52], suggesting that the genetic differentiation among landrace SPs is due to farmer trade and is mainly influenced by geographic distances. Cultivars with a CIMMYT/ICARDA origin reported lower values of gene flow than the other SPs, as reported previously by Rufo et al. [46] in bread wheat. These authors concluded that this was mainly due to the release of improved inbred lines distributed by local breeding programs through the nurseries to which these international centers distribute worldwide.

### 4.2. Detection of Selective Sweeps by EigenGWAS

Eigenvectors are frequently used to infer the genetic structure of a given population as they are estimated for any single individual. Several studies have pointed out the usefulness of primary eigenvectors to analyze population differentiation [53,54,55]. In this direction, eigenGWAS was developed by Chen et al. [13] as an approach to identify genomic regions underlying genetic differentiation. The analysis of selective sweeps produced during breeding is important for the identification of loci under selection that will be of interest for marker-assisted selection and the selection of the improved germplasm.

Other authors identified selective sweeps in hexaploid wheat. Cavanagh et al. [56] identified 21 selective regions in spring wheat and 39 in winter wheat using a worldwide collection of 2994 accessions. These authors found that most of the selective regions were involved in yield potential, vernalization, plant height, and biotic and abiotic stress. More recently, Zhou et al. [57] found 148 selective regions in a collection of 717 Chinese wheat landraces associated with yield and disease resistance. Liu et al. [15], using a worldwide panel comprising landraces from China and Pakistan and modern cultivars genotyped with the 90K SNP array, identified 477 selective sweeps. Some of these loci comprised known functional genes for disease resistance, vernalization, quality, adaptability, and yield.

This is the first study of this type conducted in durum wheat. We identified selective sweeps among Mediterranean landraces and modern cultivars in the durum wheat genome using eigenvectors as phenotypic traits in the GWAS. A total of 1575 MTAs were significant for the first ten eigenvectors at a moderate threshold, whereas for a highly significant threshold, 250 MTAs were significant. To simplify this information, 89 QTL hotspots (including 1491 MTAs) were defined as consensus genomic regions controlling loci under selection. These QTL hotspots included important loci that were selected during the breeding process such as the photoperiod loci *Ppd*-A1 and *Ppd*-B1, the vernalization loci *Vrn*-A1 and *Vrn*-B1, and the dwarfing genes *Rht*-B1, *Rht12* and *Rht25*. The cycle length of durum wheat was shortened during the breeding process by the incorporation of favorable alleles from these loci, as reported by Royo et al. [41,49]. The development of semi-dwarf germplasm by CIMMYT at the end of the 1960s had a world-wide impact on wheat production. The major dwarfing genes *Rht*-B1b and *Rht*-D1 (this last in bread wheat) incorporated in the modern cultivars reported yield increases of up to 35% in both durum wheat [50] and bread wheat [58]. The quality loci for the high molecular weight (HMW) glutenin subunits (GS) *Glu*-A1 and *Glu*-B1 were found within QTL hotspots in chromosomes 1A and 1B, respectively. Previous studies reported by De Vita et al. [59] and Subirà et al. [60] revealed the improvement of pasta-making quality in modern cultivars during the 20th century in Italy and Spain due to the incorporation of favorable alleles for HMW- and low molecular weight (LMW)-GS loci. Other loci involved in grain quality located within hotspots were the polyphenol oxidase (PPO) genes *Ppo*-A1 [61] and *Ppo*-B2 [62], which cause the undesirable brown color in semolina, and thus the identification of the alleles producing low PPO activity is essential in durum wheat breeding programs. The peroxidase activity genes, such as *Pod*-A1 [63], affect the natural carotenoid pigment content and are associated with the color of flour. *GPC*-B1, located on chromosome 6B [64], confers a shorter duration of the grain filling period due to early flag leaf senescence and thus increases grain protein content. Wheat grain avenin-like proteins (ALPs), such as *TaALP*-4A, are involved in dough quality and antifungal activities [65]. Finally, *Psy*-B1 belongs to the phytoene synthase (PSY) gene family, which are involved in the biosynthesis of carotenoid pigments in durum wheat, influencing grain yellowness [66]. Other genes included within QTL hotspots were related to grain yield, such as the locus *TaSus2*-2A which is associated with grain weight as reported by Jiang et al. [67], the transcript elongation factor *TaTEF*-7A [68] which regulates tillering and increases grain number per spike, and the glutamine synthetase gene *TaGS2*-B1 [69] which plays a key role in plant growth, nitrogen use efficiency, and yield potential in wheat. The identification of these genes that were incorporated into elite cultivars during the breeding process suggest the QTL hotspot regions as target loci in wheat improvement.

Among the 250 MTA over the highly significant threshold, 147 MTAs showed −log_10_ *p* > 5. These markers, distributed in all chromosomes except 4B, were used to perform a new PCoA. Interestingly, a similar pattern with two main groups was observed in both analyses, separating most of the landraces from modern cultivars, with a higher level of admixture among subpopulations in the latter. When markers were analyzed to find allelic differences among the two groups, five QTL hotspots (eigenQTL2A.7, eigenQTL2B.3, eigenQTL3A.5, eigenQTL3A.6, and eigenQTL3A.7) were identified as being responsible for the main separation in the PCoA among landraces and modern cultivars. However, at the genome level [21,22,23], hotspots on chromosome 3A were located in the same physical positions. Differences in the genetic position may correspond to heterozygous genotypes and missing data. According to our results, these hotspots are suggested to be the main drivers in the genetic differentiation of Mediterranean landraces from modern cultivars.

The analysis of the genome sequence covering these QTL hotspots revealed the presence of gene models involved in important biological functions (Supplementary Table S4). Among them, different gene models were related to disease resistance; a *CsAtPR5*-like protein was found to be linked to the powdery mildew resistance gene *PmLK906* in the wheat line ‘Lankao 90(6) 21-12′ [70]. According to Larriba et al. [71] Rhomboid-like proteins are involved in fungal–plant interactions. Proteins belonging to the UDP-glycosyltransferase protein superfamily were found to participate in fusarium head blight (FHB) resistance in wheat [72]. The kinase family proteins are involved in different processes, ranging from physiological roles such as control of shoots and floral meristems to pathogen identification [73]. This protein family also includes the leucine-rich repeats receptor-like kinase (LRR-RLK) genes, a large and complex gene family in plants mainly participating in the development and stress reactions. LRR domains are characterized by a high variation in the number of repeats, allowing a wide range of protein–protein interactions [74]. Proteins containing NAC and heat shock domains were reported to regulate biotic and abiotic stresses [75,76].

Other genes with implications in stress and plant development corresponded to MADS-box and tetratricopeptide repeats (TPR). According to Ma et al. [77], the MADS-box gene family plays key roles in different developmental processes such as flowering time, floral meristems, fruit formation, and flower organs and seeds. The authors found that several wheat MADS-boxes were expressed in the roots, stems, leaves, spikes, and grains during different developmental stages. Other MADS-box genes showed different expression under stress, as reported by Guo et al. [78] in response to stripe rust in wheat, suggesting their role in plant–microbe interactions. In *Brachypodium*, MADS-box genes were also identified to be regulated under drought and cold stresses [79]. TPRs mediate protein–protein interactions and are present across all plant species. Some TPRs are involved in plant stress and hormone signaling [80]. Auxin response factors (ARF) regulate the development of plant organs. Qiao et al. [81] characterized the ARF family in wheat and found that one of them, *TaARF15-A.1*, may regulate the development of roots and leaves. Expansins were found to be involved in root development. The experiments of Li et al. [82] overexpressing of the wheat expansin gene *TaEXPB23* in tobacco enhanced drought tolerance and accelerated root development. Zinc finger proteins play important roles in several plant mechanisms, from growth regulation and development, signaling and responses, to abiotic stresses. In wheat, the zinc finger protein *TaZFP34* is overexpressed in roots, reducing shoot growth but maintaining root elongation [83]. The homeobox protein LUMINIDEPEDENS was found in eigenQTL3A.5, 6, and 7 in the three genomes. This gene controls flowering time in *Arabidopsis*, as mutations in the gene have been found to produce late flowering that is partially suppressed by vernalization [84]. Other gene models within these eigenQTLs were found to enhance grain yield. F-box proteins were found in ‘Chinese Spring’ and ‘Svevo’ annotations in the three hotspots on chromosome 3A. Among the different functions of these genes, Li et al. [85] demonstrated that the F-box gene LARGER PANICLE improves the panicle architecture of rice, thus enhancing grain yield. In wheat, Hong et al. [86] reported that members of the F-box E3 ubiquitin ligases regulate spike development. Carboxypeptidases were implicated in grain size control in rice through the regulation of grain width, grain filling, and weight [87]. These authors found that the expression of GS 5 was correlated with larger grains in rice. Finally, a tapetum determinant gene was found. According to Lei and Liu [88], disrupted tapetum development alters the expression of many genes involved in male meiosis in higher plants.

## 5. Conclusions

The use of local landraces in breeding programs is considered a valuable approach to broadening the genetic background of crops lost during the breeding process and improving traits of commercial importance [40,45]. The present study uses a GWAS approach with eigenvectors to identify selective sweeps among durum wheat Mediterranean landraces and modern cultivars from different origins. Most of the chromosomes reported selective regions, some of them harboring functional genes for important agronomic traits involved in yield performance, plant development, and grain quality. Three genome regions in chromosomes 2A, 2B, and 3A were identified as the main drivers for the differentiation of the Mediterranean landraces. Within these regions, gene models for disease resistance, abiotic stress, plant development, and yield were found.

## Figures and Tables

**Table 1:** Genetic diversity and gene flow between genetic subpopulations.

Subpopulation	N	*H_T_*	*H_S_*	*D_ST_*	*G_ST_*	*Nm*
Total	388	0.40	0.37	0.03	0.08	6.02
SP 1	19	0.36	-	-	-	-
SP 2	119	0.36	-	-	-	-
SP 3	43	0.35	-	-	-	-
SP 4	116	0.40	-	-	-	-
SP 5	39	0.38	-	-	-	-
Admixed	51	0.35	-	-	-	-
SP 1–2	138	0.36	0.36	0.00	0.01	49.73
SP 1–3	62	0.33	0.35	0.02	0.07	6.90
SP 1–4	135	0.34	0.38	0.04	0.11	3.87
SP 1–5	58	0.35	0.37	0.02	0.06	7.32
SP 2–3	162	0.36	0.36	0.00	0.01	69.81
SP 2–4	235	0.40	0.38	0.01	0.03	14.41
SP 2–5	158	0.38	0.37	0.01	0.02	23.40
SP 3–4	159	0.35	0.38	0.03	0.08	5.41
SP 3–5	82	0.37	0.37	0.00	0.01	42.10
SP 4–5	155	0.34	0.39	0.06	0.16	2.54

N: number of genotypes; *H_T_*: total genetic diversity; *H_S_*: mean of genetic diversity within SPs; *D_ST_*: genetic diversity between SPs; *G_ST_*: coefficient of genetic differentiation; *Nm*: gene flow.

**Table 2:** QTL hotspots for eigenvectors.

Eigen Hotspot	CI Left	CI Right	N MTAs	FDR	Functional Genes
*eigen*QTL1A.1	12.90	40.37	58	6	
*eigen*QTL1A.2	41.38	45.57	2	0	
*eigen*QTL1A.3	75.97	86.96	13	4	
*eigen*QTL1A.4	97.58	109.75	19	5	
*eigen*QTL1A.5	116.31	119.74	3	1	*Glu-A1*
*eigen*QTL1A.6	242.61	254.18	11	0	
*eigen*QTL1B.1	31.81	36.72	10	1	
*eigen*QTL1B.2	37.42	41.32	2	0	
*eigen*QTL1B.3	42.20	52.79	15	0	
*eigen*QTL1B.4	70.87	96.28	29	1	
*eigen*QTL1B.5	96.45	115.68	15	2	
*eigen*QTL1B.6	137.22	140.22	12	0	*Glu-B1*
*eigen*QTL1B.7	195.66	202.99	4	2	
*eigen*QTL1B.8	238.34	241.34	3	0	
*eigen*QTL2A.1	10.07	14.84	2	0	*Ppd-A1*
*eigen*QTL2A.2	43.25	47.06	8	0	
*eigen*QTL2A.3	57.36	73.45	48	1	*TaSus2-2A*
*eigen*QTL2A.4	73.47	79.30	2	0	
*eigen*QTL2A.5	85.85	91.05	4	0	*Ppo-A1*
*eigen*QTL2A.6	94.09	97.09	2	0	
*eigen*QTL2A.7	112.04	126.00	101	12	
*eigen*QTL2B.1	19.94	23.26	3	0	*Ppd-B1*
*eigen*QTL2B.2	24.75	29.36	4	0	
*eigen*QTL2B.3	31.36	43.36	54	31	
*eigen*QTL2B.4	44.56	58.73	70	5	
*eigen*QTL2B.5	61.66	71.36	11	2	
*eigen*QTL2B.6	72.96	90.37	119	6	*Ppo-B2*, *TaGS2-B1*
*eigen*QTL2B.7	105.36	106.86	16	0	
*eigen*QTL3A.1	0.63	6.12	6	3	
*eigen*QTL3A.2	10.95	14.70	2	0	
*eigen*QTL3A.3	39.12	44.9	4	0	
*eigen*QTL3A.4	45.25	53.20	8	0	
*eigen*QTL3A.5	56.49	68.88	65	12	
*eigen*QTL3A.6	101.40	106.34	47	13	
*eigen*QTL3A.7	108.63	114.62	37	13	
*eigen*QTL3A.8	132.17	135.20	3	1	*Pod-A1*
*eigen*QTL3A.9	145.36	149.24	16	0	
*eigen*QTL3B.1	3.75	15.53	22	6	
*eigen*QTL3B.2	23.22	28.73	9	0	
*eigen*QTL3B.3	51.08	55.93	7	0	
*eigen*QTL3B.4	63.71	70.46	9	3	
*eigen*QTL3B.5	77.52	92.84	17	0	
*eigen*QTL3B.6	93.68	102.94	18	0	
*eigen*QTL3B.7	114.27	118.45	2	0	
*eigen*QTL3B.8	136.49	141.10	3	0	
*eigen*QTL4A.1	18.34	21.81	5	0	
*eigen*QTL4A.2	23.50	27.20	7	2	
*eigen*QTL4A.3	27.35	32.70	3	1	
*eigen*QTL4A.4	74.67	77.67	3	3	
*eigen*QTL4A.5	93.51	98.13	8	0	
*eigen*QTL4A.6	110.56	117.64	7	1	
*eigen*QTL4A.7	119.89	134.22	13	1	*TaALP-4A*
*eigen*QTL4B.1	42.86	50.32	5	0	*Rht-B1*
*eigen*QTL4B.2	73.62	77.36	2	0	
*eigen*QTL5A.1	12.37	18.03	3	0	
*eigen*QTL5A.2	33.09	38.49	6	0	
*eigen*QTL5A.3	46.29	50.38	4	0	
*eigen*QTL5A.4	56.89	67.05	6	0	
*eigen*QTL5A.5	75.81	88.74	21	2	*Vrn-A1*, *Rht12*
*eigen*QTL5A.6	104.85	116.96	10	0	
*eigen*QTL5B.1	23.38	42.70	158	51	
*eigen*QTL5B.2	51.84	56.56	2	0	
*eigen*QTL5B.3	64.79	72.95	5	0	
*eigen*QTL5B.4	83.39	86.39	3	0	*Vrn-B1*
*eigen*QTL5B.5	106.11	112.65	4	0	
*eigen*QTL5B.6	112.84	115.99	4	0	
*eigen*QTL5B.7	117.20	122.07	6	0	
*eigen*QTL5B.8	149.77	152.77	2	0	
*eigen*QTL6A.1	1.90	31.45	121	24	
*eigen*QTL6A.1	1.90	31.45	121	24	*Rht25*
*eigen*QTL6A.1	1.90	31.45	121	24	
*eigen*QTL6A.2	69.82	74.66	2	1	
*eigen*QTL6A.3	88.40	99.66	8	2	
*eigen*QTL6B.1	0.98	5.56	8	0	
*eigen*QTL6B.2	6.69	9.69	4	1	
*eigen*QTL6B.3	10.47	15.54	4	1	
*eigen*QTL6B.4	20.52	40.79	27	4	*GPC-B1*
*eigen*QTL6B.5	59.52	62.86	2	0	
*eigen*QTL6B.6	73.99	84.69	18	2	
*eigen*QTL7A.1	28.87	34.42	7	4	
*eigen*QTL7A.2	63.05	66.36	3	1	*TaTEF-7A*
*eigen*QTL7A.3	70.42	83.26	21	0	
*eigen*QTL7A.4	95.94	107.40	16	11	
*eigen*QTL7A.5	147.09	160.29	11	0	
*eigen*QTL7B.1	47.05	51.28	3	1	
*eigen*QTL7B.2	73.10	79.67	8	2	
*eigen*QTL7B.3	81.66	88.16	10	0	
*eigen*QTL7B.4	92.07	97.38	4	2	
*eigen*QTL7B.5	109.16	113.15	2	0	
*eigen*QTL7B.6	123.88	132.06	10	1	*Psy-B1*

CI: confidence interval at 95% (cM). N MTAs: number of MTAs. FDR: number of MTAs above the FDR threshold at *p* < 0.05. Functional genes co-locating with QTL hotspots were identified based on common markers with Liu et al. [15] and Pascual et al. [39].

**Table 3:** QTL hotspots involved in the selection showing allelic differences among the two PCoA groups.

QTL Hotspot	Marker	Position (cM)	Genome Position (bp)			Allele Group 1	Allele Group 2
			Zavitan	Svevo	Chinese Spring	(Frequency)	(Frequency)
*eigen*QTL2A.7	1089372	123.66	768,637,732	771,309,636	766,565,471	0 (0.81)	1 (0.82)
	1096089	123.66	768,369,404	770,792,840	767,003,197	0 (0.81)	1 (0.90)
	1288584	123.66	-	772,466,381	765,605,244	1 (0.80)	0 (0.90)
*eigen*QTL2B.3	3935165	36.35	55,282,377	53,704,532	54,005,983	0 (0.89)	1 (0.82)
	3946438	36.35	55,263,539	-	53,999,239	0 (0.84)	1 (0.87)
	3955840	36.35	55,263,539	-	53,999,239	0 (0.84)	1 (0.87)
	4404794	36.35	-	53,704,524	54,005,983	1 (1.00)	0 (0.85)
	4404891	36.35	-	53,704,524	54,005,983	1 (1.00)	0 (0.85)
	4409154	36.35	-	53,703,534	-	1 (1.00)	0 (0.85)
	3022498	37.15	56,411,136	54,740,047	55,031,700	0 (0.84)	1 (0.80)
	1125733	38.57	59,371,071	57,490,889	57,917,326	0 (0.89)	1 (0.80)
	1353553	40.74	55,744,579	54,098,441	54,443,978	C (0.84)	T (0.87)
	3021610	40.74	55,523,159	53,972,355	54,272,933	C (0.89)	T (0.87)
	4004228	40.74	57,503,553	56,011,661	55,991,662	1 (0.95)	0 (0.85)
	4004312	40.99	56,411,136	54,740,047	55,031,700	1 (0.95)	0 (0.82)
	986135	40.99	56,166,013	54,516,891	54,786,611	A (0.89)	C (0.85)
	1124640	41.86	56,147,572	54,468,610	54,770,824	A (0.84)	G (0.85)
*eigen*QTL3A.6	2257732	103.85	693,610,895	688,415,545	697,202,220	0 (0.98)	1 (0.98)
	1007286	103.92	687,773,343	682,345,965	691,736,154	0 (0.98)	1 (0.95)
	1061286	103.92	687,959,611	682,871,589	692,054,958	0 (0.99)	1 (0.88)
	1099726	103.92	693,660,065	-	697,248,312	0 (0.98)	1 (0.97)
	2257138	103.92	688,886,018	683,409,098	692,987,209	0 (0.98)	1 (0.99)
	3033940	103.92	690,079,348	684,307,722	694,092,980	0 (0.96)	1 (0.99)
	3940178	103.92	691,844,961	686,017,151	695,739,301	0 (0.97)	1 (0.99)
	3945420	103.92	688,521,622	682,907,278	692,471,812	0 (0.98)	1 (0.96)
	3952975	103.92	688,369,820	685,647,294	692,316,203	0 (0.98)	1 (0.98)
	3957848	103.92	691,844,961	686,017,151	695,739,301	0 (0.97)	1 (0.99)
	4005072	103.92	688,885,643	683,409,473	692,987,584	0 (0.97)	1 (0.99)
*eigen*QTL3A.7	1062254	110.13	691,603,242	685,647,297	695,515,284	T (0.98)	G (0.98)
	1120615	110.13	687,953,731	682,789,499	692,048,455	1 (0.95)	0 (0.96)
	1127998	110.13	691,772,662	685,990,476	695,671,629	T (0.93)	C (0.96)
	1755023	110.13	692,894,801	687,945,767	-	1 (0.99)	0 (0.96)
	2275425	110.13	690,565,280	684,664,645	694,538,535	A (0.98)	G (0.97)
	4003435	110.13	689,966,914	684,172,863	693,979,130	1 (0.99)	0 (0.96)
	4004625	110.13	-	682,650,634	-	1 (0.98)	0 (0.97)

The markers showed −log_10_ *p* > 5. PAV: presence/absence variant; SNP: single nucleotide polymorphism.

## Data Availability

All data generated during this study are included in this published article (and its Supplementary Information files).

## Supplementary Materials

The following are available online at https://www.mdpi.com/2079-7737/10/4/258/s1, Figure S1: Polymorphic information content distribution among markers. Darker bar indicates the average PIC value; Figure S2: Manhattan plots for the 10 eigenvectors; Table S1: List of cultivars used in the study. SP: Subpopulation; q: membership coefficient for the SP; Table S2: EigenGWAS results; Table S3: Analysis of marker alleles between groups 1 and 2 in the PCoA which resulted from the 147 significant markers with –lop_10_ *p* > 5, and among SPs in group 1 and 2; Table S4: Gene models identified within the QTL hotspots eigenQTL2A.7, eigenQTL2B.3 and eigenQTL3A.5–7.
