# Supplementary material for: Labelling Selective Sweeps Used in Durum Wheat Breeding from a Diverse and Structured Panel of Landraces and Cultivars
Source: Biology (Basel). 2021 Mar 24;10(4):258. doi: 10.3390/biology10040258 (PMC8064341; doi:10.3390/biology10040258)
Supplement: Supplementary file 1 [file biology-10-00258-s001.zip › biology-1139514- Figure S1-S2-Table S1.docx]

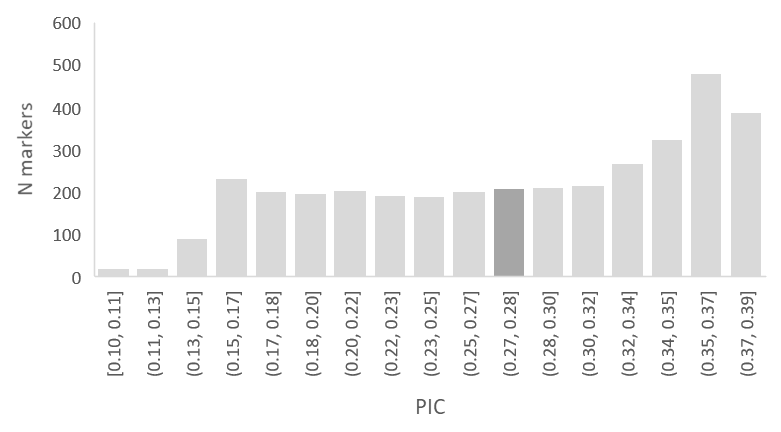


**Figure S1.** Polymorphic information content distribution among markers. Darker bar indicates the average PIC value.

Eigenvector 1


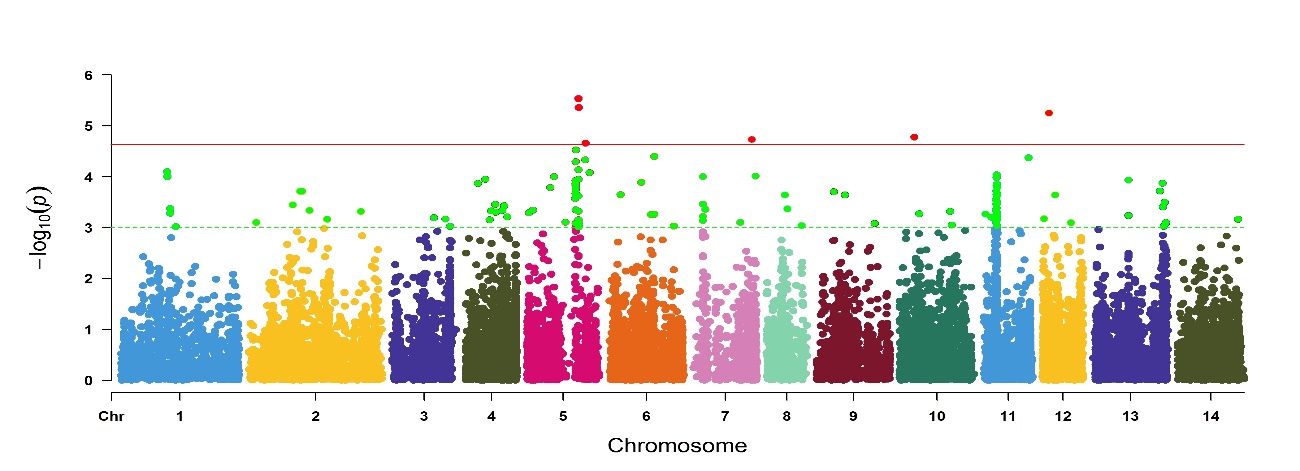


Eigenvector 2


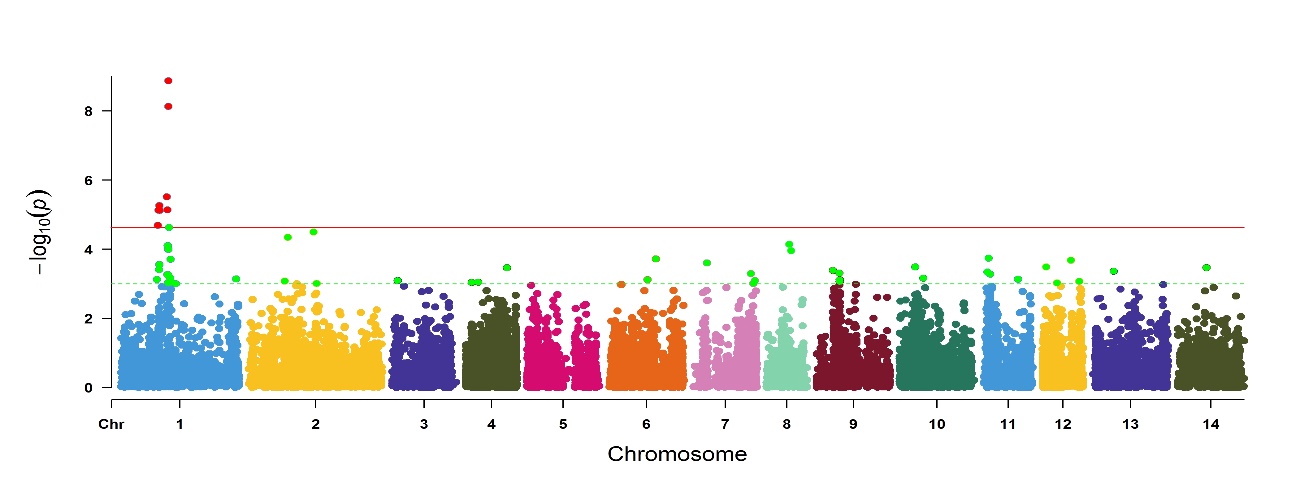


Eigenvector 3


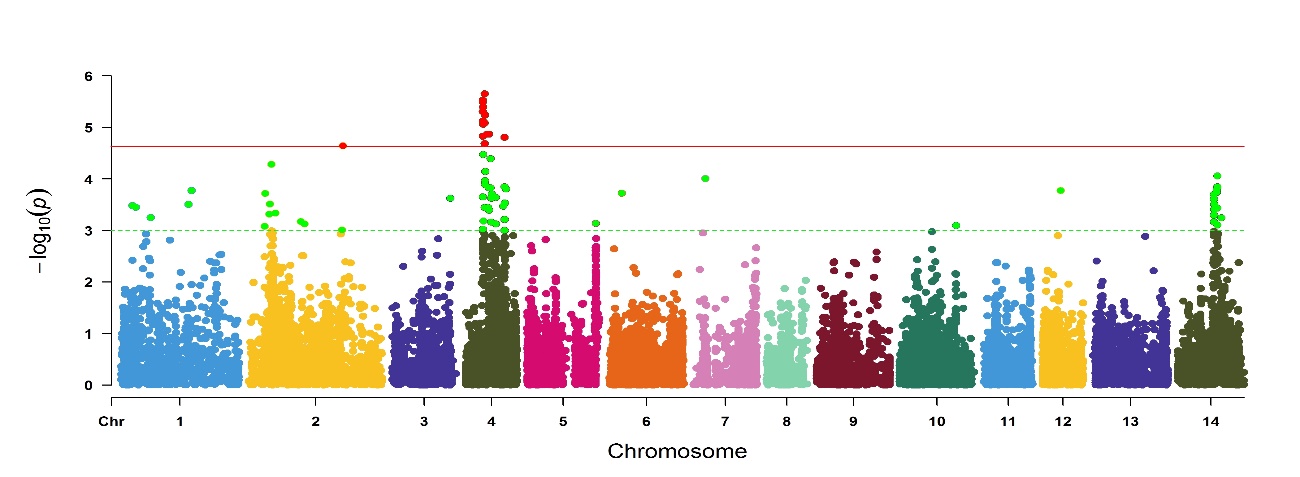


Eigenvector 4


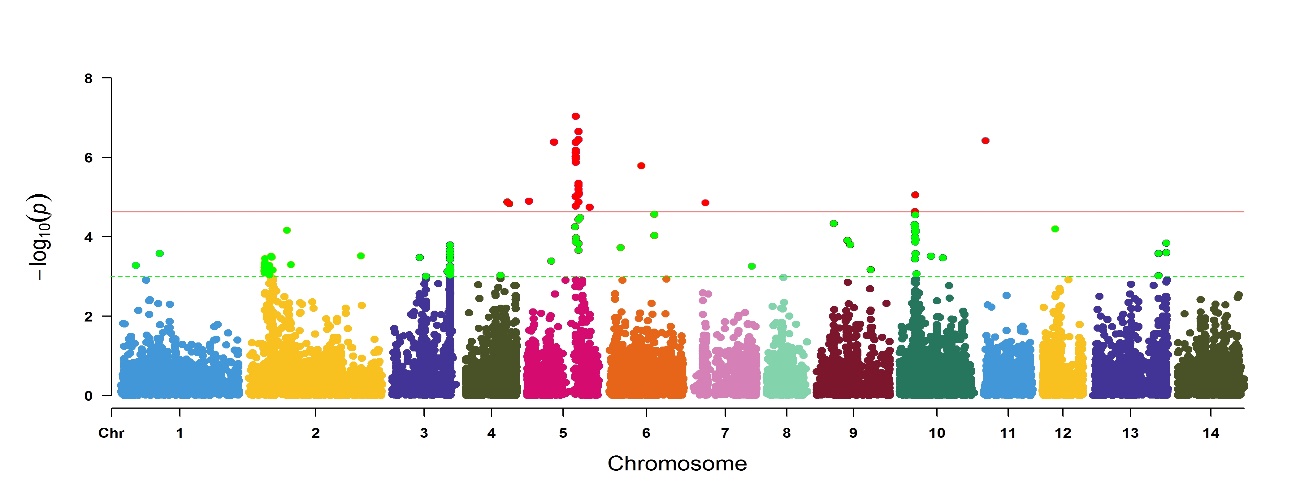


Eigenvector 5


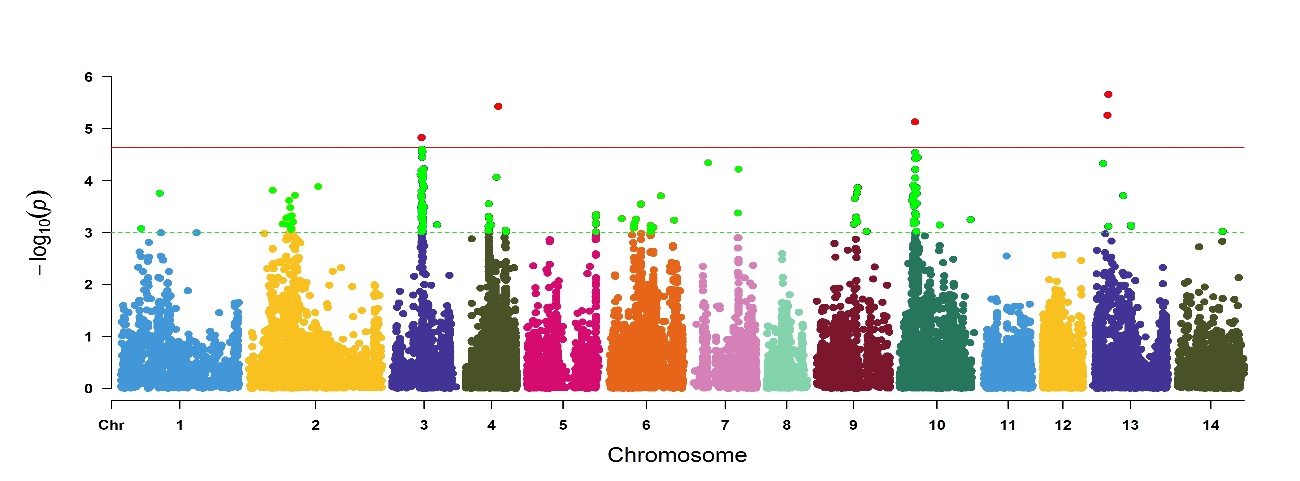


Eigenvector 6


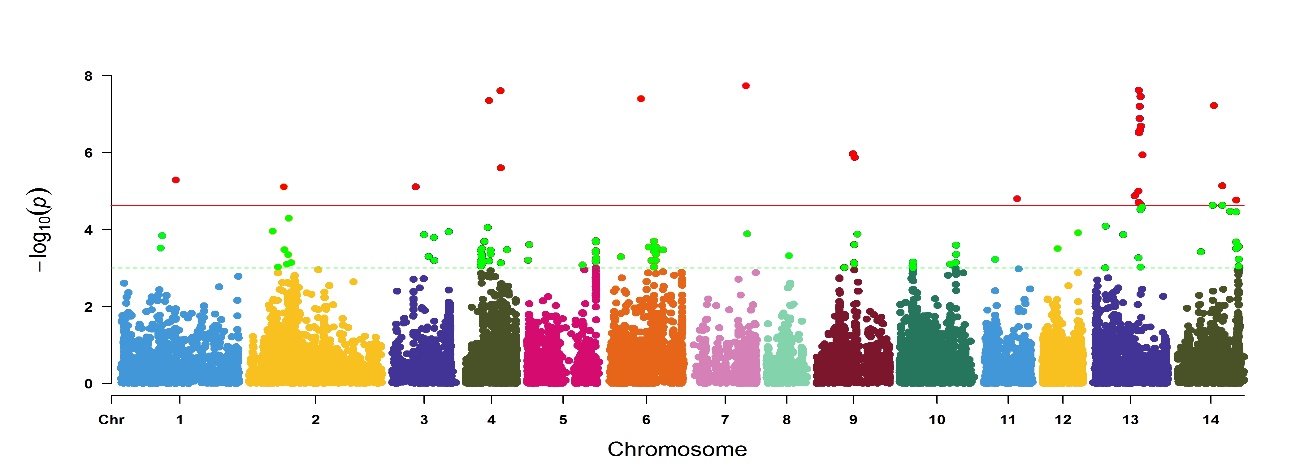


Eigenvector 7


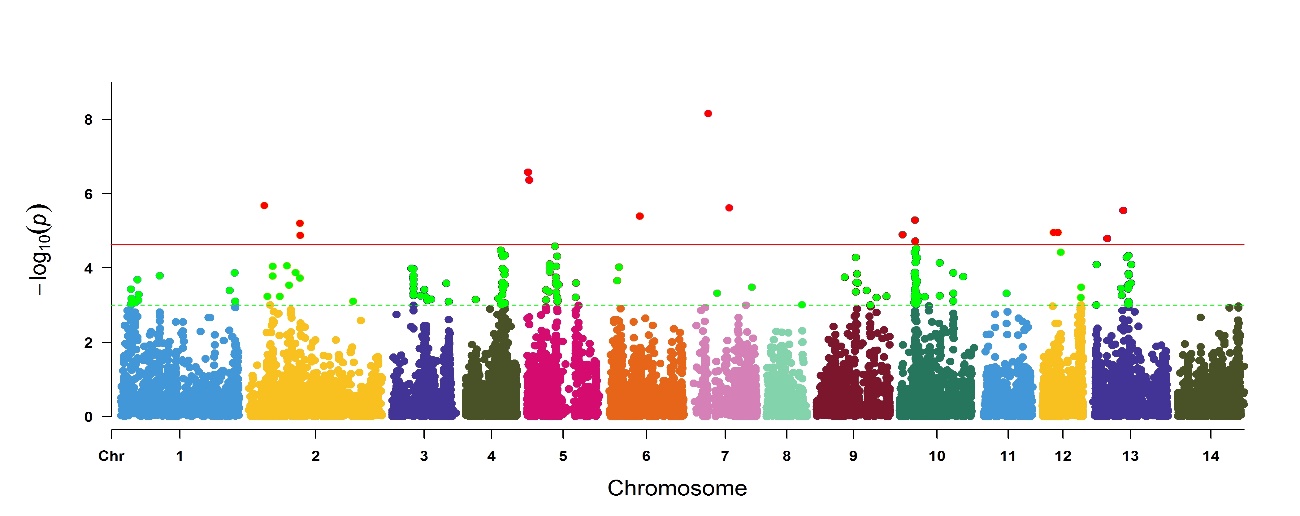


Eigenvector 8


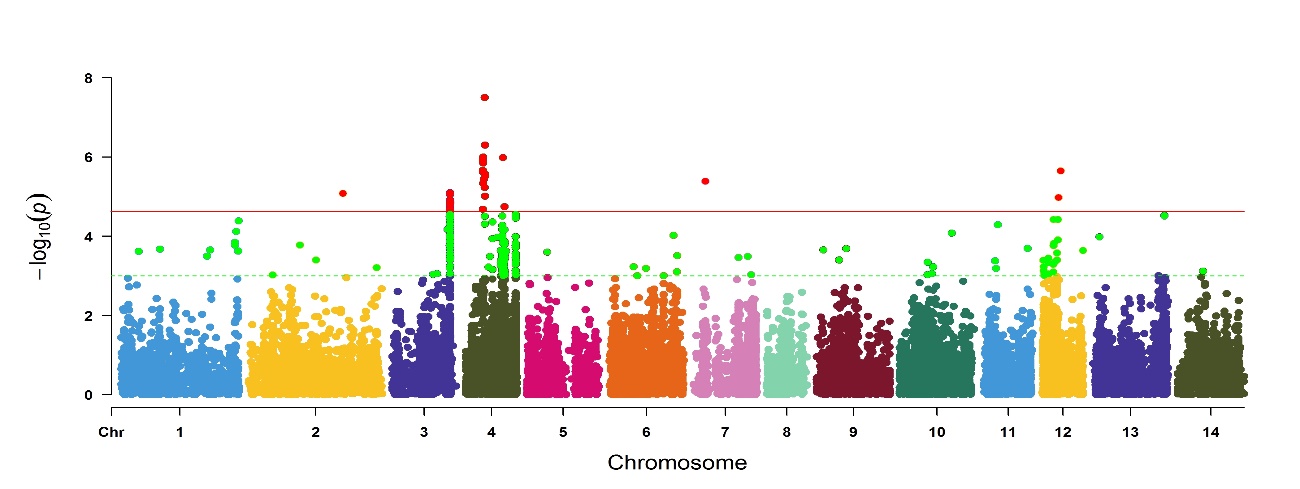


Eigenvector 9


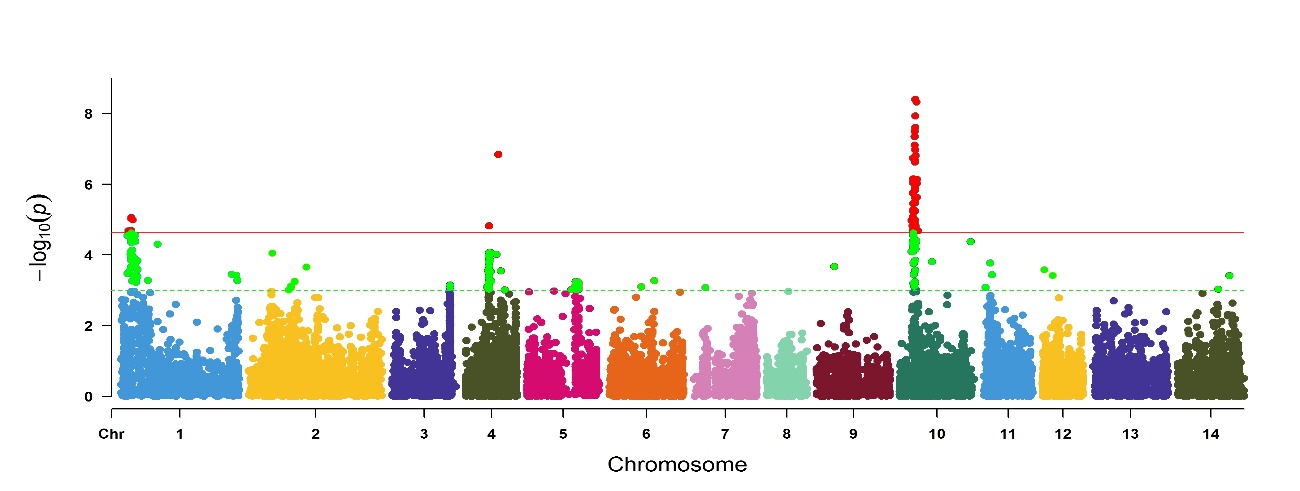


Eigenvector 10


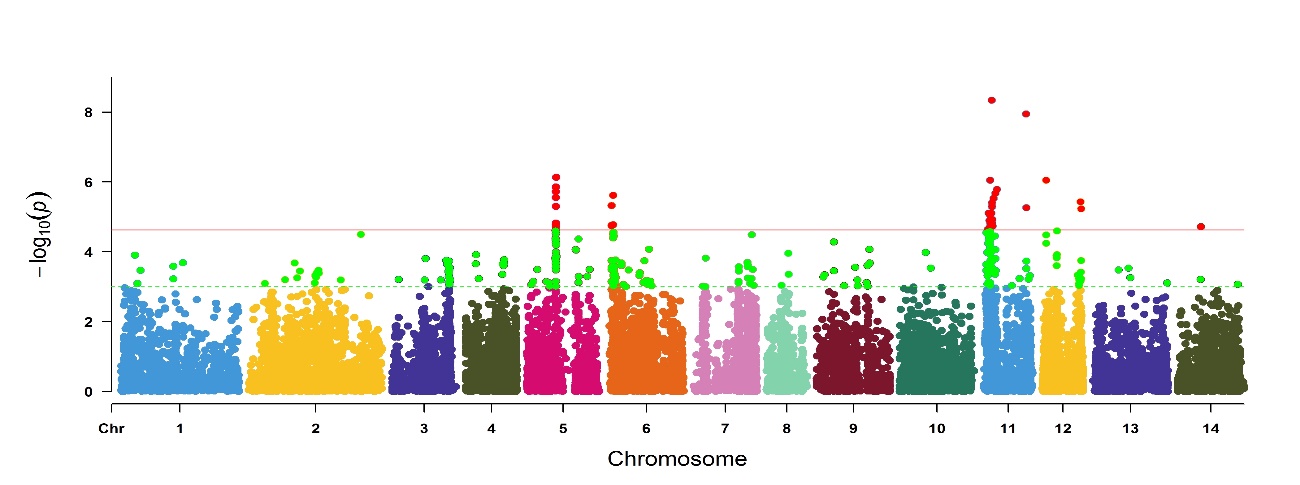


**Figure S2.** Manhattan plots for the 10 eigenvectors.

**Table S1.** List of cultivars used in the study. SP: Subpopulation; q: membership coefficient for the SP.

| **Cultivar** | **Country** | **Type** | **SP** | **q** |
| --- | --- | --- | --- | --- |
| IG-92895 | Algeria | Landrace | Admixed | - |
| IG-92967 | Algeria | Landrace | SP3 | 0.537 |
| IG-93030 | Algeria | Landrace | SP3 | 0.991 |
| IG-93621 | Algeria | Landrace | SP3 | 0.658 |
| IG-94009 | Algeria | Landrace | SP2 | 0.646 |
| Dur de Medeah | Algeria | Landrace | SP2 | 0.73 |
| Tchirpan | Bulgaria | Landrace | SP2 | 0.586 |
| Lozen 76 | Bulgaria | Landrace | Admixed | - |
| Zagorka | Bulgaria | Landrace | SP2 | 0.556 |
| IG-96802 | Crete | Landrace | SP3 | 0.633 |
| IG-96851 | Crete | Landrace | SP2 | 0.626 |
| Dalmatia 1 | Croatia | Landrace | SP2 | 0.805 |
| Dalmatia 3 | Croatia | Landrace | SP2 | 0.84 |
| 440-IX/96 | Croatia | Landrace | SP2 | 0.895 |
| 441-IX/97 | Croatia | Landrace | SP2 | 0.883 |
| Vroulos | Cyprus | Landrace | SP2 | 0.689 |
| IG-82549 | Cyprus | Landrace | SP2 | 0.677 |
| Muri | Cyprus | Landrace | SP2 | 0.665 |
| Akathiotico Naurotheri | Cyprus | Landrace | SP2 | 0.74 |
| FAO 29.912 | Cyprus | Landrace | SP2 | 0.668 |
| Pansyiotico | Cyprus | Landrace | SP2 | 0.653 |
| Kambourico Famaquita | Cyprus | Landrace | SP3 | 0.524 |
| Kyperounda Yiallouriko | Cyprus | Landrace | SP2 | 0.64 |
| Milagro | Egypt | Landrace | SP2 | 0.582 |
| Reading | Egypt | Landrace | SP2 | 0.541 |
| D-2 | Egypt | Landrace | Admixed | - |
| 5P4 | Egypt | Landrace | SP2 | 0.519 |
| 1P1 | Egypt | Landrace | SP2 | 0.534 |
| Giza 2 | Egypt | Landrace | Admixed | - |
| 2751 | Egypt | Landrace | Admixed | - |
| MG 26429 | Egypt | Landrace | Admixed | - |
| 28 | Egypt | Landrace | Admixed | - |
| 31 | Egypt | Landrace | Admixed | - |
| Sinai No.8 | Egypt | Landrace | SP1 | 0.672 |
| Mishriki | Egypt | Landrace | SP2 | 0.631 |
| Girgeh | Egypt | Landrace | Admixed | - |
| Beladi Rouge | France | Landrace | SP2 | 0.75 |
| De Santa Marta | France | Landrace | SP2 | 0.73 |
| Iumillo | France | Landrace | SP2 | 0.574 |
| Tounse | France | Landrace | SP2 | 0.625 |
| Trigo Glutinoso | France | Landrace | SP2 | 0.546 |
| Rubio enlargado d’Atlemteje | France | Landrace | SP2 | 0.695 |
| Mavraani | Greece | Landrace | SP2 | 0.755 |
| Rapsani | Greece | Landrace | SP2 | 0.686 |
| Greece 14 | Greece | Landrace | SP2 | 0.596 |
| Greece 23 | Greece | Landrace | SP2 | 0.59 |
| Greece 24 | Greece | Landrace | SP2 | 0.748 |
| Iran 1 | Iran | Landrace | SP2 | 0.802 |
| Abu Fashit | Israel | Landrace | SP2 | 0.549 |
| Etith | Israel | Landrace | SP1 | 0.998 |
| Juljulith | Israel | Landrace | SP2 | 0.601 |
| Hati | Israel | Landrace | SP1 | 0.784 |
| JM-3987 | Israel | Landrace | Admixed | - |
| JM-3989 | Israel | Landrace | Admixed | - |
| Carlantino | Italy | Landrace | SP2 | 0.522 |
| Cicirelo | Italy | Landrace | SP2 | 0.738 |
| IG-83905 | Italy | Landrace | SP2 | 0.605 |
| IG-83920 | Italy | Landrace | SP1 | 0.907 |
| Carlo jucci | Italy | Landrace | SP3 | 0.792 |
| Senatore Capelli | Italy | Landrace | SP3 | 0.999 |
| Hymera | Italy | Landrace | SP3 | 0.589 |
| Trinakria | Italy | Landrace | SP3 | 0.71 |
| Aziziah 17/45 | Italy | Landrace | SP1 | 0.999 |
| Razza 208 | Italy | Landrace | SP3 | 0.992 |
| Balilla Falso | Italy | Landrace | SP2 | 0.697 |
| Milazzo | Italy | Landrace | SP2 | 0.639 |
| Razza 181 | Italy | Landrace | SP2 | 0.747 |
| Capeiti | Italy | Landrace | SP3 | 0.546 |
| Razza 96 | Italy | Landrace | SP3 | 0.999 |
| Capeiti 8 | Italy | Landrace | SP3 | 0.584 |
| Safra Jerash | Jordan | Landrace | SP1 | 0.677 |
| Harani Auttma | Jordan | Landrace | SP1 | 0.999 |
| Salti na Zinia | Jordan | Landrace | SP2 | 0.587 |
| Horani Howawi | Jordan | Landrace | SP1 | 0.997 |
| Zugbieh Sutra | Jordan | Landrace | SP1 | 0.998 |
| Zoghbiyeh Safra | Jordan | Landrace | SP1 | 0.977 |
| Safra Maan | Jordan | Landrace | SP1 | 0.998 |
| 26 | Jordan | Landrace | SP1 | 0.934 |
| IG-84856 | Lebanon | Landrace | Admixed | - |
| 9923 | Lebanon | Landrace | SP1 | 0.966 |
| 9929 | Lebanon | Landrace | SP1 | 0.999 |
| 9935 | Lebanon | Landrace | SP1 | 0.908 |
| 9918 | Lebanon | Landrace | Admixed | - |
| Hourah | Lebanon | Landrace | SP1 | 0.981 |
| Reyati | Lebanon | Landrace | SP3 | 0.6 |
| Tripshiro | Libya | Landrace | SP1 | 0.735 |
| 248-VII/7 | Macedonia | Landrace | SP2 | 0.763 |
| 259-VII/12 | Macedonia | Landrace | SP2 | 0.806 |
| VII/13-X11 | Macedonia | Landrace | SP2 | 0.801 |
| 196/71 | Macedonia | Landrace | SP2 | 0.795 |
| II/4 | Macedonia | Landrace | SP2 | 0.8 |
| 1640 | Macedonia | Landrace | SP2 | 0.733 |
| II/10 | Macedonia | Landrace | SP3 | 0.682 |
| 356-I/9 | Montenegro | Landrace | SP2 | 0.861 |
| 23 | Montenegro | Landrace | SP2 | 0.883 |
| 33 | Montenegro | Landrace | SP2 | 0.856 |
| 37 | Montenegro | Landrace | SP2 | 0.886 |
| 42 | Montenegro | Landrace | SP2 | 0.892 |
| Zoco Yebel Hebil | Morocco | Landrace | SP2 | 0.599 |
| Maghoussa | Morocco | Landrace | SP3 | 0.513 |
| Merzaga | Morocco | Landrace | SP3 | 0.722 |
| Red Beard | Morocco | Landrace | SP3 | 0.923 |
| Morocco | Morocco | Landrace | SP3 | 0.737 |
| Saffi | Morocco | Landrace | SP3 | 0.746 |
| Ble Dur 250 | Morocco | Landrace | SP3 | 0.594 |
| Oned Zenati | Morocco | Landrace | SP3 | 0.992 |
| Mahmoudi C | Morocco | Landrace | SP3 | 0.998 |
| Maghoussa Amizmiz | Morocco | Landrace | SP2 | 0.532 |
| Cobros | Morocco | Landrace | SP2 | 0.683 |
| Haj Mouline | Morocco | Landrace | Admixed | - |
| Douro Boukowo | Morocco | Landrace | SP2 | 0.862 |
| Du Maroc Battandier | Morocco | Landrace | SP3 | 0.563 |
| Marques | Portugal | Landrace | SP2 | 0.548 |
| Raposinho | Portugal | Landrace | SP2 | 0.732 |
| Durazio Rijo | Portugal | Landrace | SP2 | 0.675 |
| Raspinegro | Portugal | Landrace | SP2 | 0.555 |
| Anafil | Portugal | Landrace | SP2 | 0.801 |
| Espanhol | Portugal | Landrace | SP2 | 0.575 |
| Dezassete | Portugal | Landrace | SP2 | 0.802 |
| Durazio Rijo Glabro | Portugal | Landrace | SP2 | 0.519 |
| Amarelo Barba Preta | Portugal | Landrace | SP2 | 0.573 |
| Alentejo | Portugal | Landrace | SP2 | 0.84 |
| Caxudo de sete espigas | Portugal | Landrace | SP2 | 0.525 |
| Tremes rijo | Portugal | Landrace | SP2 | 0.645 |
| Lobeiro de grao escuro | Portugal | Landrace | SP2 | 0.687 |
| D-1995 | Russia | Landrace | SP2 | 0.514 |
| IC 7640 | Russia | Landrace | SP3 | 0.999 |
| Belgrade 9 | Serbia | Landrace | SP2 | 0.852 |
| 1575 | Serbia | Landrace | SP2 | 0.774 |
| 18/71 | Serbia | Landrace | SP2 | 0.648 |
| Arisnegro de Tenerife | Spain | Landrace | Admixed | - |
| Basto Duro | Spain | Landrace | SP2 | 0.738 |
| Blanco de Corella | Spain | Landrace | SP2 | 0.858 |
| Blanquillo | Spain | Landrace | SP2 | 0.868 |
| Candeal de Salamanca | Spain | Landrace | SP2 | 0.543 |
| Colorado de Jerez | Spain | Landrace | SP2 | 0.712 |
| Enano de Andújar | Spain | Landrace | SP3 | 0.536 |
| Fartó | Spain | Landrace | Admixed | - |
| Griego de Baleares | Spain | Landrace | SP2 | 0.867 |
| Gros de Cerdaña | Spain | Landrace | SP2 | 0.869 |
| Heraldo del Rhin | Spain | Landrace | SP2 | 0.833 |
| Pinet | Spain | Landrace | SP2 | 0.649 |
| Pisana cañihueca | Spain | Landrace | SP2 | 0.854 |
| Raspinegro Canario | Spain | Landrace | SP2 | 0.72 |
| Raspinegro de Alcalá Guadaira | Spain | Landrace | SP2 | 0.661 |
| Recio de Almería | Spain | Landrace | SP3 | 0.798 |
| Verdial | Spain | Landrace | SP2 | 0.644 |
| Alonso | Spain | Landrace | SP2 | 0.518 |
| Andalucía 344 | Spain | Landrace | SP2 | 0.518 |
| Azulejo de Villa del Río | Spain | Landrace | SP2 | 0.692 |
| Blancal | Spain | Landrace | SP3 | 0.801 |
| Blanquillón de Boñar | Spain | Landrace | SP2 | 0.804 |
| Claro de Balazote | Spain | Landrace | SP2 | 0.784 |
| Entrelargo de Montijo | Spain | Landrace | SP2 | 0.738 |
| Farto cañifino | Spain | Landrace | SP2 | 0.768 |
| Rubio de Montijo | Spain | Landrace | SP2 | 0.717 |
| Semental | Spain | Landrace | SP2 | 0.592 |
| Recio de Cañete | Spain | Landrace | SP3 | 0.768 |
| Rubio de Belalcázar | Spain | Landrace | SP2 | 0.774 |
| Blanco Verdeal | Spain | Landrace | SP2 | 0.582 |
| Clarofino | Spain | Landrace | SP2 | 0.762 |
| IG-95812 | Syria | Landrace | SP1 | 0.993 |
| IG-95841 | Syria | Landrace | SP2 | 0.501 |
| IG-95847 | Syria | Landrace | SP1 | 0.999 |
| IG-95931 | Syria | Landrace | Admixed | - |
| Haurani 79-b | Syria | Landrace | SP2 | 0.587 |
| Louri AP 5 | Tunisia | Landrace | SP2 | 0.725 |
| Souri | Tunisia | Landrace | SP2 | 0.65 |
| Realforte | Tunisia | Landrace | SP3 | 0.632 |
| Biskri | Tunisia | Landrace | SP3 | 0.994 |
| Hamira | Tunisia | Landrace | SP2 | 0.51 |
| Jennah Khetifa Rp4 | Tunisia | Landrace | SP2 | 0.697 |
| Mindium | Turkey | Landrace | SP2 | 0.583 |
| BGE-018192 | Turkey | Landrace | SP2 | 0.739 |
| BGE018351 | Turkey | Landrace | SP2 | 0.595 |
| BGE018353 | Turkey | Landrace | SP2 | 0.671 |
| BGE-018354 | Turkey | Landrace | SP2 | 0.766 |
| BGE019262 | Turkey | Landrace | SP2 | 0.742 |
| BGE-019263 | Turkey | Landrace | SP2 | 0.803 |
| BGE019264 | Turkey | Landrace | SP2 | 0.744 |
| BGE019265 | Turkey | Landrace | SP2 | 0.76 |
| BGE019266 | Turkey | Landrace | SP2 | 0.791 |
| BGE-019270 | Turkey | Landrace | SP2 | 0.55 |
| SAHEL 77 | Algeria | Modern | SP4 | 0.599 |
| BONAERENSE VALVERDE | Argentina | Modern | SP4 | 0.936 |
| BUCK CANDISUR | Argentina | Modern | SP4 | 0.528 |
| BUCK CRISTAL | Argentina | Modern | SP4 | 0.984 |
| ARIVATO | Australia | Modern | Admixed | - |
| BELLAROI | Australia | Modern | Admixed | - |
| KALKA | Australia | Modern | Admixed | - |
| SAINTLY | Australia | Modern | Admixed |  |
| TAMAROI | Australia | Modern | SP4 | 0.587 |
| Ocotillo | Canada | Modern | SP5 | 0.872 |
| Macoun | Canada | Modern | SP3 | 0.999 |
| Wakooma | Canada | Modern | SP5 | 0.881 |
| Waskana | Canada | Modern | SP5 | 0.929 |
| AC AVONLEA | Canada | Modern | SP5 | 0.843 |
| AC MORSE | Canada | Modern | SP5 | 0.993 |
| AC NAVIGATOR | Canada | Modern | SP5 | 0.978 |
| AC PATHFINDER | Canada | Modern | SP5 | 0.951 |
| COMMANDER | Canada | Modern | SP5 | 0.981 |
| STRONGFIELD | Canada | Modern | SP5 | 0.641 |
| CHAGUAL INIA | Chile | Modern | SP4 | 0.999 |
| CHONTA INIA | Chile | Modern | SP4 | 0.755 |
| GUAYACAN INIA | Chile | Modern | SP4 | 0.725 |
| QUILAFEN | Chile | Modern | SP4 | 0.691 |
| UCARO 1 | Chile | Modern | SP4 | 0.754 |
| Gallareta | CIMMYT | Modern | SP4 | 0.973 |
| Jupare | CIMMYT | Modern | SP4 | 0.917 |
| Sula | CIMMYT | Modern | SP4 | 0.998 |
| Vitron | CIMMYT | Modern | SP4 | 0.998 |
| Somat | CIMMYT | Modern | SP4 | 0.844 |
| CIMMYT 67 - Plata 16 | CIMMYT | Modern | SP4 | 0.999 |
| CIMMYT 73 - Porto 5 | CIMMYT | Modern | SP4 | 0.997 |
| ARONAS | Cyprus | Modern | Admixed | - |
| MESAORIA | Cyprus | Modern | SP4 | 0.602 |
| ARENDETO | Ethiopia | Modern | SP4 | 0.618 |
| BOOHAI | Ethiopia | Modern | SP3 | 0.502 |
| HORA | Ethiopia | Modern | SP4 | 0.808 |
| MAROU | Ethiopia | Modern | SP4 | 0.92 |
| Arment | France | Modern | Admixed | - |
| Arcalis | France | Modern | Admixed | - |
| Arcodur | France | Modern | SP5 | 0.721 |
| Aronde | France | Modern | SP3 | 0.61 |
| Artimon | France | Modern | SP4 | 0.841 |
| Attila | France | Modern | SP4 | 0.59 |
| Auroc | France | Modern | SP5 | 0.949 |
| Epidur | France | Modern | SP5 | 0.512 |
| Flodur | France | Modern | SP5 | 0.606 |
| Bonitec | Germany | Modern | SP4 | 0.999 |
| Burgos | Germany | Modern | SP4 | 0.598 |
| MALAVIKA | India | Modern | SP4 | 0.776 |
| MALVARAJ | India | Modern | SP4 | 0.646 |
| NARBADA 215 | India | Modern | SP4 | 0.755 |
| RAJ 1555 | India | Modern | SP4 | 0.728 |
| WH 896 | India | Modern | SP4 | 0.913 |
| OSCAR | Iran | Modern | Admixed | - |
| HAZERA | Israel | Modern | SP4 | 0.795 |
| Claudio | Italy | Modern | SP4 | 0.616 |
| Meridiano | Italy | Modern | SP4 | 0.817 |
| Simeto | Italy | Modern | SP3 | 0.624 |
| Svevo | Italy | Modern | SP4 | 0.789 |
| Creso | Italy | Modern | SP2 | 0.78 |
| Flavio | Italy | Modern | Admixed | - |
| Ambral | Italy | Modern | SP5 | 0.676 |
| Anento | Italy | Modern | SP3 | 0.64 |
| Ardente | Italy | Modern | Admixed | - |
| Casiello | Italy | Modern | SP3 | 0.566 |
| Fenice | Italy | Modern | SP4 | 0.751 |
| Appulo | Italy | Modern | SP3 | 0.517 |
| Fortore | Italy | Modern | Admixed |  |
| Adamello | Italy | Modern | Admixed | - |
| Cirillo | Italy | Modern | SP5 | 0.665 |
| Zenit | Italy | Modern | SP5 | 0.797 |
| Annouar | Morocco | Modern | SP4 | 0.999 |
| Karim | Morocco | Modern | SP4 | 0.999 |
| Massa | Morocco | Modern | SP4 | 0.95 |
| Ouedezena | Morocco | Modern | SP4 | 0.999 |
| Sarif | Morocco | Modern | SP4 | 0.683 |
| Tassaout | Morocco | Modern | SP4 | 0.685 |
| Yasmine | Morocco | Modern | SP4 | 0.697 |
| 1804 | Morocco | Modern | SP4 | 0.622 |
| 1805 | Morocco | Modern | SP4 | 0.849 |
| 1807 | Morocco | Modern | SP4 | 0.68 |
| 1808 | Morocco | Modern | SP4 | 0.665 |
| 1809 | Morocco | Modern | SP4 | 0.898 |
| ENDURO | Netherland | Modern | SP4 | 0.55 |
| WADHANAK 85 | Pakistan | Modern | SP4 | 0.904 |
| Alcamin | Portugal | Modern | Admixed | - |
| Bakht | Russia | Modern | Admixed | - |
| Selinogradskaja | Russia | Modern | Admixed | - |
| Amilcar | Spain | Modern | SP4 | 0.997 |
| Ancalei (PNTD/1) | Spain | Modern | SP4 | 0.951 |
| Astigi | Spain | Modern | SP4 | 0.789 |
| Boabdil | Spain | Modern | SP4 | 0.726 |
| Bolido | Spain | Modern | SP4 | 0.623 |
| Bolo | Spain | Modern | SP4 | 0.702 |
| Hispasano (PNTD/3) | Spain | Modern | SP4 | 0.892 |
| Senadur | Spain | Modern | SP5 | 0.584 |
| Vitronero | Spain | Modern | SP4 | 0.537 |
| Mexa | Spain | Modern | SP5 | 0.534 |
| Camacho | Spain | Modern | SP3 | 0.558 |
| Bidi 17 | Spain | Modern | SP3 | 0.999 |
| Abadia | Spain | Modern | Admixed | - |
| Anibal | Spain | Modern | SP2 | 0.502 |
| Donduro | Spain | Modern | SP4 | 0.809 |
| Asdrúbal | Spain | Modern | SP5 | 0.783 |
| Belladur | Spain | Modern | SP4 | 0.598 |
| Bonzo | Spain | Modern | Admixed | - |
| Boreal | Spain | Modern | SP4 | 0.699 |
| Borgia | Spain | Modern | SP3 | 0.535 |
| Carpio | Spain | Modern | SP4 | 0.544 |
| Debano | Spain | Modern | Admixed | - |
| Duradero | Spain | Modern | SP4 | 0.991 |
| Excalibur | Spain | Modern | SP5 | 0.508 |
| Grecale | Spain | Modern | Admixed | - |
| Imhotep | Spain | Modern | Admixed | - |
| Jabato | Spain | Modern | Admixed | - |
| Jaguar | Spain | Modern | SP4 | 0.543 |
| Jiloca | Spain | Modern | SP3 | 0.554 |
| Kidur | Spain | Modern | SP5 | 0.744 |
| Lebrija | Spain | Modern | SP3 | 0.718 |
| Mellaria | Spain | Modern | Admixed | - |
| Mexidur | Spain | Modern | Admixed | - |
| Paramo | Spain | Modern | SP2 | 0.665 |
| Pingüino | Spain | Modern | SP4 | 0.538 |
| Ponferrada | Spain | Modern | SP4 | 0.634 |
| Prospero | Spain | Modern | SP4 | 0.738 |
| Ramirez | Spain | Modern | SP4 | 0.781 |
| Randur | Spain | Modern | SP5 | 0.521 |
| Safari | Spain | Modern | SP5 | 0.678 |
| Santadur | Spain | Modern | SP4 | 0.783 |
| Semolero | Spain | Modern | SP4 | 0.784 |
| Severo | Spain | Modern | SP4 | 0.922 |
| Taranto | Spain | Modern | SP4 | 0.96 |
| Tejón | Spain | Modern | SP4 | 0.531 |
| Tetradur | Spain | Modern | SP5 | 0.793 |
| Valgera | Spain | Modern | Admixed | - |
| Valira | Spain | Modern | SP4 | 0.728 |
| Esquilache | Spain | Modern | Admixed | - |
| Ariesol | Spain | Modern | SP5 | 0.58 |
| Euroduro | Spain | Modern | SP4 | 0.871 |
| Awalbit-7 | Syria | Modern | Admixed | - |
| Brachoua | Syria | Modern | SP4 | 0.897 |
| Chahba 88 | Syria | Modern | SP4 | 0.931 |
| Chanst | Syria | Modern | SP4 | 0.855 |
| Fardes | Syria | Modern | SP4 | 0.888 |
| Massara-1 | Syria | Modern | Admixed | - |
| Moosabil-1 | Syria | Modern | Admixed | - |
| Omrabi 5 | Syria | Modern | Admixed | - |
| Sabil 1 | Syria | Modern | SP4 | 0.998 |
| Stojocri-2 | Syria | Modern | SP4 | 0.567 |
| Stork | Syria | Modern | SP5 | 0.552 |
| Aghrass-1 | Syria | Modern | SP4 | 0.701 |
| Ammar-1 | Syria | Modern | SP4 | 0.936 |
| Arislahn-5 | Syria | Modern | SP4 | 0.768 |
| Awali-1 | Syria | Modern | SP4 | 0.718 |
| Bicre | Syria | Modern | SP4 | 0.985 |
| Chacan | Syria | Modern | SP4 | 0.793 |
| Cham-1 | Syria | Modern | Admixed | - |
| Derra | Syria | Modern | SP4 | 0.615 |
| Guerou-1 | Syria | Modern | SP4 | 0.95 |
| Kabir1 | Syria | Modern | SP4 | 0.673 |
| Khabur-1 | Syria | Modern | SP4 | 0.893 |
| Lagonil-2 | Syria | Modern | SP4 | 0.718 |
| Lagost 3 | Syria | Modern | SP4 | 0.739 |
| Lahn | Syria | Modern | SP4 | 0.991 |
| Loukos-1 | Syria | Modern | SP4 | 0.957 |
| Maamouri-1 | Syria | Modern | SP4 | 0.849 |
| Marsyr-1 | Syria | Modern | SP4 | 0.841 |
| Moulsabil 2 | Syria | Modern | SP4 | 0.85 |
| Murlagost-1 | Syria | Modern | SP4 | 0.778 |
| Omgenil-3 | Syria | Modern | SP4 | 0.712 |
| Omrabi 3 | Syria | Modern | Admixed | - |
| Omruf-2 | Syria | Modern | SP4 | 0.958 |
| ORT-1 | Syria | Modern | SP4 | 0.695 |
| Ouaserl-1 | Syria | Modern | SP4 | 0.679 |
| Ouasloukos-1 | Syria | Modern | SP4 | 0.916 |
| Quabrach-1 | Syria | Modern | SP4 | 0.828 |
| Sebah | Syria | Modern | SP4 | 0.738 |
| Stojocri-3 | Syria | Modern | SP4 | 0.571 |
| Terbol97-3 | Syria | Modern | SP4 | 0.779 |
| Wadalmez-1 | Syria | Modern | SP4 | 0.774 |
| Zeina 1 | Syria | Modern | SP4 | 0.678 |
| Waha | Syria | Modern | Admixed | - |
| KARIM 80 | Tunisia | Modern | SP4 | 0.999 |
| KHIAR 92 | Tunisia | Modern | SP4 | 0.997 |
| MÂALI | Tunisia | Modern | SP4 | 0.858 |
| NASR 99 | Tunisia | Modern | SP4 | 0.694 |
| RAZZAK 87 | Tunisia | Modern | SP4 | 0.844 |
| AMANOS 97.3.1 | Turkey | Modern | SP4 | 0.691 |
| Kronos | USA | Modern | SP5 | 0.533 |
| Duraking | USA | Modern | SP4 | 0.679 |
| Fjord | USA | Modern | SP5 | 0.996 |
| Lakota | USA | Modern | SP5 | 0.615 |
| Lloyd | USA | Modern | SP5 | 0.884 |
| Matt | USA | Modern | SP5 | 0.795 |
| Medora | USA | Modern | SP5 | 0.998 |
| Modoc | USA | Modern | Admixed | - |
| Monroe | USA | Modern | SP5 | 0.997 |
| Orita | USA | Modern | SP5 | 0.587 |
| Vic | USA | Modern | SP5 | 0.941 |
| Ward | USA | Modern | SP5 | 0.998 |
| West Bred Laker | USA | Modern | SP5 | 0.581 |
| Colorado | USA | Modern | SP5 | 0.862 |
| Cortez | USA | Modern | Admixed | - |
| Durex | USA | Modern | SP5 | 0.772 |
| West Bred Turbo | USA | Modern | SP4 | 0.681 |
